# Progress of Dental Surgery

**Published:** 1846-12

**Authors:** 


### PROGRESS OF DENTAL SURGERY.

We have lately received the first annual catalogue of the Ohio College of Dental Surgery, in Cincinnati. It has a board of trustees acting under a charter from the Legislature of that State, and a respectable Faculty, including professors of Anatomy and Chemistry. The three professors of Dental Science and Art, are not inferior in skill and attainment to any others in the Valley of the Mississippi. The college has erected a suitable edifice and furnished it with the requisite means of instruction. The class of last year was decidedly encouraging. We cannot but look with warm approbation and lively hope upon this first Western effort to create a school for the education of dental surgeons; and we trust that the day is not distant, when no dentist will receive the patronage of society who is not regularly educated.

We have also received the "Proceedings of the Mississippi Valley Association of Dental Surgeons," at their second annual meeting, held at Cincinnati, in the Hall of the College, in the month of September last. One object of this Association is, to create a profession, and dignify it, by adopting high rules of professional conduct. Another, is to elicit papers that shall communicate valuable discoveries; thereby provoking that discussion which cannot fail to develop truth, correct error, and quicken invention. These objects are of the highest importance. We perceive by the "Proceedings" that the Association numbers about thirty members. It should comprehend at least a hundred; and we hope ere long to hear of such an augmentation. It is time that those who practise this branch of the healing art should awake from their slumber, and leave their insulation, and look each other in the face, and take each other by the hand, and make "a long pull, a strong pull, and a pull all together," to advance and elevate that portion of *the* profession which is confided to them.

